# Naphthalene or Mothball Poisoning Manifesting as Acute Intravascular Hemolysis and Acquired Methemoglobinemia

**DOI:** 10.7759/cureus.64325

**Published:** 2024-07-11

**Authors:** Salman Habib Roghani, Mohammad Ali Arif, Rauf Niazi, Sadia Mansoor

**Affiliations:** 1 Internal Medicine, Pakistan Institute of Medical Sciences, Islamabad, PAK

**Keywords:** mothball, naphthalene poisoning, n-acetyl cystine, ascorbic acid, methylene blue, methemoglobinemia, intravascular hemolysis, naphthalene

## Abstract

Naphthalene is a major component of mothballs. Domestically, people use mothballs as an insect repellent. Its deliberate or accidental ingestion leading to toxicity has rarely been reported in the medical literature, despite its widespread use in Southeast Asia. Naphthalene, or mothball poisoning, is a rare but serious condition that can have detrimental effects on human health. This case report presents the clinical course of a 22-year-old male who ingested six naphthalene balls, resulting in severe symptoms including fever, abdominal pain, vomiting, jaundice, and dark-colored urine. Laboratory investigations were suggestive of acute intravascular hemolysis and methemoglobinemia. The patient was promptly admitted to the hospital, where he received supportive care along with specific treatment in the form of red blood cell transfusions, intravenous methylene blue, ascorbic acid, and N-acetyl cysteine. Through this report, the importance of raising awareness about the dangers of naphthalene poisoning and the specific treatment options available is highlighted.

## Introduction

Acute poisoning is one of the most frequent presentations to emergency departments across Pakistan. Cases frequently seen are paracetamol, benzodiazepine, organophosphorus, opioid, and wheat pill (aluminum phosphide) poisonings. Naphthalene or mothball poisoning is a rare occurrence. In Pakistan, people commonly use naphthalene, a simple polycyclic aromatic hydrocarbon, as an insect repellent in the form of mothballs. It is readily available in all shops and supermarkets. One mothball is 4 grams in weight. The toxic dose for adults is 5-15 grams and 2-3 grams for children [1]. Overdose may result from oral ingestion, inhalation, or prolonged skin exposure. The common presenting symptoms of naphthalene toxicity are fever, headache, vomiting, diarrhea, abdominal pain, jaundice, dark-colored urine, and confusion. The complications are acute intravascular hemolysis, resulting in severe anemia and increased leucocyte count, methemoglobinemia, and acute kidney injury [2]. Methemoglobinemia results in a decrease in red blood cells' ability to carry oxygen, leading to potentially life-threatening situations [3]. We present the case of a 22-year-old Pakistani male who, after deliberate ingestion of six naphthalene balls, developed complications of acquired intravascular hemolysis and methemoglobinemia. By shedding light on this specific case, we aim to underscore the importance of education and awareness in preventing similar instances and ultimately improving patient care outcomes.

## Case presentation

A 22-year-old male with no previous co-morbidities presented to the emergency department after ingestion of six mothballs (naphthalene balls) with the intention of self-harm. He presented with a one-day history of fever, headache, abdominal pain, yellow discoloration of the skin, and black urine. His vital signs were stable and within normal limits. A physical examination revealed that he was markedly pale and had jaundice. The systemic examination was unremarkable.

Initial investigations (Table 1) revealed severe anemia with marked leucocytosis, a normal platelet count, and a normal coagulation profile. Clinical jaundice, elevated total bilirubin, elevated reticulocyte count, and elevated lactate dehydrogenase (LDH) all suggested intravascular hemolysis. Hemoglobinuria was confirmed by a positive urine analysis. Liver function tests, renal function tests, and creatine phosphokinase (CPK) were normal. The glucose-6-phosphate dehydrogenase (G6PD) assay was normal.

**Table 1:** Progression of biochemical parameters Hb: hemoglobin; LDH: lactate dehydrogenase

Lab Parameters	Day 1	Day 2	Day 3	Day 4	Day 5	Day 6	Reference Range
Hb g/dl	4.2	5.7	11.7	13.7	13.5	13.5	11-18
Platelets/ul	148000	156000	191000	167000	158000	157000	150000-400000
Total bilirubin mg/dl	6.6	4.6	3.1	1.0	1.5	0.5	0.3-1.2
LDH U/L	2241	1645	1131	950	739	349	50-460
Urine colour	Black	Black	Amber	Amber	Amber	Amber	Clear/Amber
Blood in urine	3+	2+	1+	Nil	Nil	Nil	Nil
Met Hb levels	Sample sent	Positive	Negative	Negative	Negative	Negative	Negative

Gastric lavage was done with activated charcoal as per hospital guidelines. He was treated with a rapid infusion of normal saline and a transfusion of packed red cell concentrate. N-acetyl cysteine (600 mg three times a day (TDS)) and ascorbic acid (500 mg once a day (OD)) were also given. Methemoglobin levels were sent.

Subsequently, the patient became drowsy and cyanosed, his Glasgow Coma Scale (GCS) dropped, and he was not maintaining saturation on room air. Arterial blood gas showed a normal partial pressure of oxygen (PaO2). These findings were suggestive of methemoglobinemia. The patient was intubated and transferred to the intensive care unit (ICU) for mechanical ventilation. During his stay in the ICU, he was aggressively hydrated with normal saline and was given methylene blue 1 mg/kg (total dose 50 mg) 1% solution in normal saline over five to 30 minutes. Daily transfused with packed red cell concentrate. The patient remained hemodynamically stable, and his biochemical parameters improved day by day, as shown in Table 1. The patient's condition significantly improved during hospitalization, resulting in a comfortable discharge.

## Discussion

Naphthalene is a solid polycyclic hydrocarbon that is obtained from coal tar or crude oil through a process called fractional distillation. It is widely used as an insect repellent. Toxicity is associated with hemolytic anemia, liver abnormalities, acute kidney injury, neurological symptoms like confusion and convulsions, and ocular effects like cataracts and retinal hemorrhage. Also classified as a carcinogen, naphthalene increases the risk of developing head, neck, and gastrointestinal cancers [4].

Naphthalene enhances the production of free radicals, which cause oxidative stress and subsequently damage to cells due to lipid peroxidation [5]. Individuals who are vulnerable to oxidative stress can present with hemolysis, such as people with G6PD deficiency [6]. G6PD has a critical role in red cell metabolism because it prevents oxidative stress on the cell and, thus, reduces the severity of naphthalene toxicity [7].

Naphthalene toxicity causes oxidative stress, which results in methemoglobinemia. In methemoglobin, the iron (Fe) in the haem group is in the ferric (Fe +3) state, not the ferrous (Fe +2). Methaemoglobin cannot bind oxygen, and this shifts the oxygen-dissociation curve to the left, causing tissue hypoxia. Clinically, this is observed as central cyanosis, which occurs at methemoglobin levels above 1.5 g/dL [8]. Co-oximetry will help in its diagnosis, as pulse oximetry is not accurate in such patients [9].

Treatment of naphthalene toxicity is mainly supportive [10]. Underlying co-morbidities, including cardiac, respiratory, and hematologic diseases, may lead to adverse outcomes [11]. Ascorbic acid is used in doses of 300 mg as a treatment to decrease the oxidative stress of naphthalene [12,13]. Methylene blue is the main treatment for methemoglobinemia. It increases the rate of reduction of methoglobin to hemoglobin. The recommended dose is 1-1.5 mg/kg body weight for adults via a slow intravenous infusion [14]. A G6PD assay should be performed prior to administration, as methylene blue itself can cause hemolysis in patients with G6PD deficiency, leading to paradoxical methemoglobinemia. N-acetylcysteine (NAC) is a reducing agent and can also be used in the treatment of methemoglobinemia. Exchange transfusion is an alternative option. Continuous renal replacement therapy can be considered in cases of acute kidney injury or to eliminate toxins.

## Conclusions

In conclusion, the general population should be educated and made aware of these dangerous chemicals and their handling. Health authorities, with the help of policymakers, should form a national Toxbase for all poisonings. This Toxbase already exists in various developed countries and contains all the information related to different poisonings. There are no proper guidelines for dealing with naphthalene poisoning. The main guiding factors are the severity and mode of presentation. The mainstay of treatment is red blood cell transfusion, intravenous methylene blue, N-acetylcysteine, ascorbic acid, and, in some cases, renal replacement therapy. Clinicians should be aware of the different presentations and complications of naphthalene poisoning, as it is an extremely common item in households.
